# The aesthetics of frieze patterns: Effects of symmetry, motif, and element size

**DOI:** 10.1177/20416695221131112

**Published:** 2022-10-27

**Authors:** Jay Friedenberg, Preston Martin, Naomi Uy, Mackenzie Kvapil

**Affiliations:** 2010 Manhattan College, New York, NY, USA; 12292 University at Buffalo, Buffalo, NY, USA; 2010 Manhattan College, New York, NY, USA; 2010 Manhattan College, Riverdale, NY, USA

**Keywords:** features/parts, perception, perceptual organization, texture, symmetry, aesthetics, friezes, beauty

## Abstract

Frieze patterns appear frequently in architectural designs and ornamental patterning but their aesthetic qualities have never been studied experimentally. In the first experiment, 39 undergraduates used a seven-point rating scale to assess the perceived beauty of the seven basic frieze types presented at a horizontal orientation. The friezes consisted of individual curved and linear motifs as well as random textures. Friezes that filled the entire pattern region and which contained emergent global features were preferred the most. In a second experiment, we utilized horizontal texture friezes that were completely filled and which varied in size and number of elements. Participants preferred patterns with larger features, probably because they make detection of the symmetric transformations more visible. The frieze with the greatest number of symmetries was preferred most but symmetric complexity by itself could not completely account for the predicted preference ordering. In both studies, friezes containing horizontal mirrors (translation, 180° rotation, horizontal mirror, vertical mirror, and glide reflection and translation, horizontal mirror, and glide reflection) were preferred far more than any other condition. Horizontal symmetry may enhance perceived beauty in these cases because it runs parallel to and so emphasizes the overall frieze orientation.

## The Aesthetics of Frieze Patterns: Effects of Symmetry and Motif

The purpose of decorative art is to adorn and aesthetically enhance the surface of everyday objects (Kirkman & Weber, 2013; Miller, 2006). The decorative arts are different from fine art, in which the art “object” such as a painting or photograph is primary and considered unto itself. Decoration, or ornament as it is sometimes also called, has received relatively little research attention compared to the fine arts but that is hopefully now changing (Whitfield & de Destefani, 2011). Ornamentation is important because it is widespread in our everyday experiences (Weil & Weil, 2009). One needs only to look around to see that we are constantly exposed to these patterns in the form of architecture, textiles, graphic design, etc. In contrast, we experience the fine arts much less often and usually in a location specifically designed for it like a museum or concert hall.

Border patterns or friezes are one type of decorative art. A frieze is formed by the repetition of a basic motif or pictorial element in one direction. The mathematical analysis of these patterns has led to a classification of frieze types based on group theory. There are seven types of frieze, each characterized by symmetry that maps one motif onto another. The set of symmetries present in a frieze type defines a frieze group. All seven friezes are present in the art of cultures worldwide and through most recorded historical periods (Stevens, 1981). Examples can be found in Indian ceramics and baskets of the American southwest, cloths from Zaire and Nigeria, Persian rugs, Peruvian textiles, and Chinese bronze containers (Washburn & Crowe, 1988). Some issues have yet to be addressed concerning the aesthetic appreciation of friezes. Are some preferred over others? Is this related to their type and number of symmetries? Is the kind of motif or the number of elements in a frieze related to their perceived beauty? In this study, we examine how symmetry and complexity affect the perceived beauty of friezes.

Friezes are linear transformations in one direction and as such are considered one-dimensional (Grunbaum & Shephard, 1989). Each repetition of the motif has to be the same size and shape as the original. There are only some allowed moves or transformations, called isometries or rigid transformations. Isometries do not alter the motif in terms of its length, angles, or area. There are four possible isometries in the plane: translation, reflection, rotation, and glide reflection. Translation along a line repositions a motif parallel to its original location. Reflection about a line or axis produces a mirror image of the motif. Rotation spins the motif about a point, and glide reflection is the combination of a linear translation and reflection.

**Figure 1.**
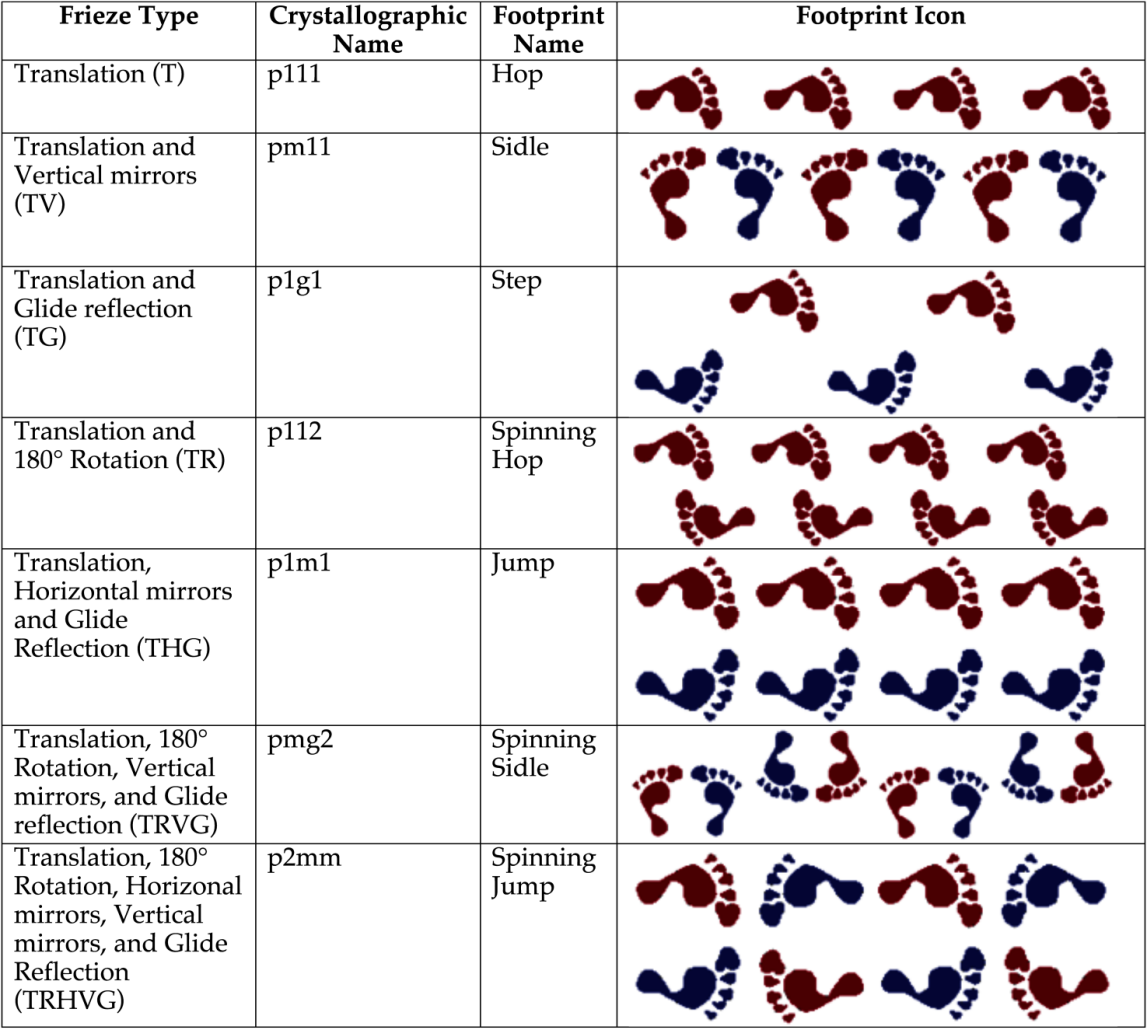
Frieze type, crystallographic name, footprint name, and footprint icon for each of the seven frieze types. A description of the naming convention is provided. The first character in this naming convention is p, standing for primitive cell. The second character is m if there is a mirror line perpendicular to the center line of the strip and 1 otherwise. The third character is m if there is a mirror line along the center line of the strip, g if there is not a mirror line but there is a glide reflection along the center line of the strip, and 1 if there is no reflection or glide reflection along the center line. The fourth character is 2 if there is a 180° rotation and 1 if there is not.

Figure 1 shows examples of the various frieze types and different naming conventions. The frieze T (p111) is the simplest and contains only translational symmetry defined by the repetition of a motif. The next set of three friezes has two types of symmetry. Frieze translation plus vertical mirror (TV) (pm11) contains translation plus vertical mirror symmetry. Here each of the motifs is reflected about a vertical axis. In frieze translation plus glide reflection (TG) (p1g1) we have translation and glide reflection. In this case, the elements are translated and then reflected about a horizontal axis. Frieze translation and 180° rotation (TR) (p112) have translation and 180° rotation. For frieze translation, horizontal mirror, and glide reflection (THG) (p1m1) there are now three kinds of symmetry, translation, horizontal mirrors in which the elements are mirror images reflected about a horizontal axis and glide reflection. In frieze Translation, 180° rotation, Vertical mirror, and Glide reflection (TRVG) (pmg2) there are four symmetries: translation, rotation, vertical mirrors, and glide reflection. The seventh and final frieze translation, 180° rotation, horizontal mirror, vertical mirror, and glide reflection (TRHVG) (p2mm) is the most complex, containing all five symmetries: translation, rotation, horizontal and vertical mirrors, and glide reflection. One way to help think about the friezes is in terms of the impression of footprints (Conway, 1992). An alternate crystallographic nomenclature exists and is listed in Figure 1 (Kinsey & Moore, 2002).

Most of the research on friezes comes from mathematics and computer science, where their symmetric properties have been analyzed. An important step in the computational processing of a frieze is symmetry detection. This is an important aspect of recognition algorithms because symmetry is present in so many objects and textures. Programs have been created that can detect symmetry in natural images (Liu et al., 2010). This ability aids object recognition and natural scene understanding. Applications include face analysis, vehicle detection, and medical image analysis (Kuehnle, 1991; Mancas et al., 2005; Mitra & Liu, 2004). The detection of symmetry in frieze and wallpaper patterns is a precondition for these algorithms and can help provide insight into how the human visual system may perform such calculations.

Liu and Collins (1998) developed an algorithm capable of classifying patterns in terms of the seven frieze groups and 17 two-dimensional wallpaper patterns. It was tested on computer-generated images and photos of natural scenes and extended to work on two-dimensional patterns that are distorted under affine transformations. Liu et al. (2004) later developed a computational model to recognize and classify periodic patterns based on crystallographic groups. It worked by automatically finding the underlying lattice, identifying the symmetry group, and extracting the motifs. There are a number of applications of such models, including texture synthesis, image compression, and gait analysis.

However, symmetry as it occurs in nature is not always of the strictly straight type found in decorative friezes. It is often curved. Examples of this include leaves and animal bodies. Lee and Liu (2012) created an algorithm capable of dealing with this situation. It could detect curved glide reflection with relative success in a large data set of leaf images. While linear symmetries may more often be the norm in decorative art and objects of an artifactual nature, curved glide reflection is more frequently a property of natural objects. It is unclear whether it processed or responded to difference in human perception.

There has been little work in experimental psychology on the topic of ornamental art or friezes. Most of the research on decoration examines order versus randomness and symmetry. Kaili et al. (2019) had participants evaluate square-shaped decorative patterns using the implicit association task. They found that regular patterns were preferred over random ones. Their regular stimuli were arrowhead-shaped motifs arranged in a structured square array with translational symmetry and the same patterns at different random orientations without any symmetry. The findings suggest that translation enhances the processing fluency of decorative patterns, which may account for their increased aesthetic quality.

Another recent study using frieze-like stimuli also finds an order preference. Westphal-Fitch and Fitch (2017) had participants rate bench tile patterns with translational, reflectional and rotational symmetry (low entropy scores) and those with random patterning (high entropy scores). Observers preferred symmetric tiles. In a separate task, participants were asked to produce these types of tiles in terms of what they or others might like. The production task resulted in the formation of fewer symmetric patterns than those on the benches. Translation was present more in both the perception and production tasks even though reflectional symmetry is generally more salient for human perception and beauty judgments (Jacobsen & Höfel, 2002).

The studies cited above suggest that observers usually prefer regularity. However, this might not always be the case. Friedenberg (2017) found reflection was preferred to the rotation for elongated contour polygons but that there was no difference in preference between the translation and random shapes. This latter result suggests that symmetry is not always preferred over asymmetry. One reason for this may have to do with complexity. Translation can be considered the simplest of all symmetries and in some cases randomness could be preferred because translation is *too* simple and boring. Reflection may also not be always preferred in experts, again because of its predictability (Leder et al., 2019).

There is a long history of studies of the relationship between complexity and preference (Birkhoff, 1933; Eisenman & Rappaport, 1967; Nadal et al., 2010; Van Geert & Wagemans, 2020). Frieze patterns provide an interesting opportunity to study the effects of complexity and symmetry together. The different friezes vary in terms of the number and type of symmetric operations they contain. They provide a test case for what symmetries may be considered appealing when they are combined with one another. For example, one can study whether the addition of glide reflection in pattern TG is preferred to its absence in pattern T. Alternatively, one can look at whether the addition of horizontal mirrors in THG is preferred to its absence in frieze TG. The seven friezes vary in terms of whether they contain one, two, three, four or five varieties of symmetry. A preference for symmetric complexity would predict that beauty ratings would go up with an increase in these symmetries.

Another way that complexity can be explored is through the type of motif. Some friezes are characterized by a single motif separated from their partners. Others fill up the entire rectangular region. The former are more object-like, and the latter are more texture or surface-based. Individual motifs may be considered simpler but the symmetric relations between them may be more obvious. Texture motifs with more elements can be considered more complex. They also contain parts that group together to form larger emergent features that may enhance the perception of symmetry. Complexity can additionally be studied in texture friezes by varying the total number of elements. If complex patterns are preferred, then those with more elements might be judged more beautiful. In this study, we examine how symmetry and complexity affect the perceived beauty of friezes. Frieze type allows us to look at how the overall number and addition of different kinds of symmetries affects aesthetic judgment. Motif allows us to examine the effect of individual versus texture style elements and the presence of emergent grouping.

## Experiment 1

### Method

#### Participants

Forty undergraduates participated to fulfill a class requirement. There were 22 males and 18 females. The vision was normal or corrected to normal. Average age was 18.92 years. All participants agreed to participate and signed a consent form. American Psychological Association ethical standards and data confidentiality were adhered to.

#### Stimuli

**Figure 2.**
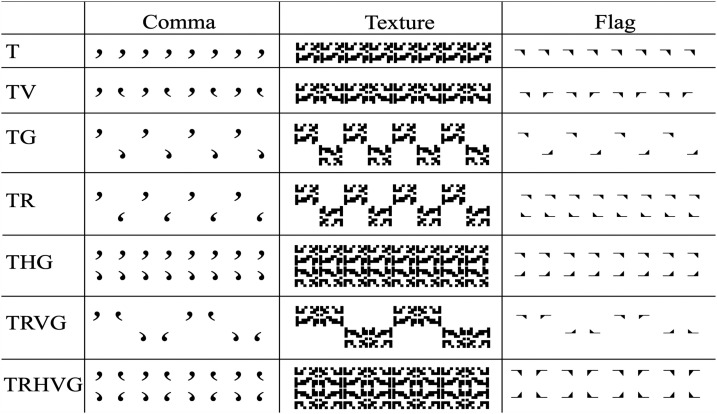
The frieze types and motifs used in Experiment 1.

The seven frieze patterns were labeled according to their type of symmetry. There was Translation (T), TV, TG, TR, THG, TRVG, and TRHVG. Three different versions of each of these seven basic pattern types were created using a different motif, either a curved comma shape, a linear flag shape, or a square block containing a random texture. Examples of the friezes used in the study are shown in Figure 2. Motifs were black against a white background. All materials used to conduct the research will be made available to other researchers for purposes of replicating the procedure or reproducing the results.

The overall region for the friezes was a rectangular area measuring 5 cm by 20 cm that ran horizontally across the screen. Each motif was centered in a square region or block within this space that measured 2.5 cm in width. The T and TV patterns were “single layer,” containing one strip of motifs. The remaining patterns were “double layered,” containing two strips of motifs, one above the other. Only the THG and TRHVG patterns filled the entire frieze space. Size of the comma and flag motifs was the same.

The third version of the stimuli was a texture block consisted of an 8 × 8 array of smaller square-sized areas measuring 0.31 cm. Each of these was filled with a 0.5 probability to generate a 50% density region. This single identical pattern was used in all of the texture friezes. In patterns where texture blocks were adjacent to another a small 0.10 cm space was inserted. This was done to prevent the formation of obvious emergent features at border regions. We wanted each texture block to be perceived as a single patterned motif so that it was perceptually equivalent to the other motifs and could be compared to them.

#### Procedure

Each of the seven friezes with their three motifs resulted in 21 unique stimuli. This constituted a single block of trials. Order of presentation within a block was randomized. Participants viewed ten blocks in an experimental session, a total of 210 trials. They were given as much time as they needed to respond. On average, the experiment took about 20 min to complete. Stimuli were presented at a standard viewing distance of 48 cm. Rating judgments were obtained for each trial.

Beauty judgments were made using the number scale that ran across the top of the computer keyboard. This scale ran from 1 to 7, with “1” labeled as *a Little Beautiful,* “4” labeled as *Average Beauty*, and “7” labeled as *Most Beautiful*. Participants were instructed to use any number from the entire scale. They were additionally told there was no right or wrong answer and that they could rate the patterns in any way they wanted. This was done to reduce any experiment-induced judgment criteria or demand characteristics. If any number other than 1–7 was entered the participant would not be able to advance to the next trial. If this occurred they were instructed to re-enter an appropriate value.

### Results

A two-way analysis of variance (ANOVA) with alpha level set at 0.05 yielded a main effect for Motif (Flag, Comma, Texture), *F* (2, 117) = 18.83, *p* < .001 (η^2^_p_ = 0.04), and Frieze Type (T, TR, TV, TG, THG, TRVG, and TRHVG), *F* (6, 273) = 42.67, *p* < .001, (η^2^_p_ = 0.24). Additionally, there was a small but significant interaction between Motif and Frieze Type, *F* (12, 819) = 2.27, *p* = .007, (η^2^_p_ = 0.03). The texture motif was rated significantly more beautiful than the comma motif or the flag motif. Next, we looked at mean beauty ratings for each frieze type. Frieze TRHVG (with all of the symmetries, including horizontal reflection) was the highest rated, followed by THG (only the second such type to contain horizontal reflection symmetry). It is probably not an accident that these two friezes were rated much higher than the others. In these patterns the reflection axis runs parallel to the overall pattern orientation. This may make the symmetries present more salient.

**Figure 3.**
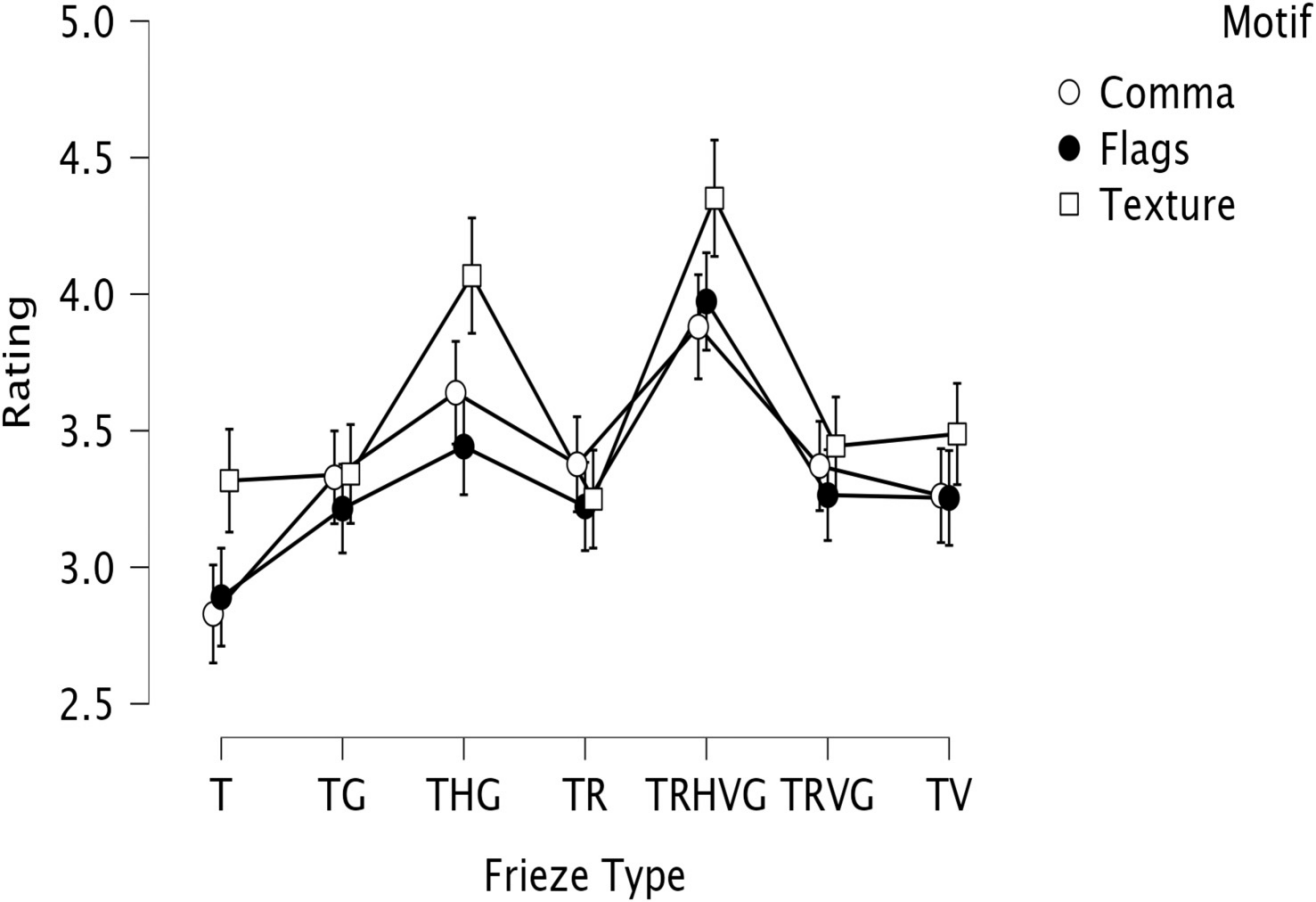
Mean and 95% confidence interval plots for the main effects of motif, frieze type and their interaction from Experiment 1.

Following these two types were the friezes TG, TR, and TV (translation plus one other type of symmetry) and TRVG which were all equivalent statistically. Frieze T (with only translation) was rated least beautiful. Figure 3 shows the interaction. Table 1 shows the Tukey Least Squares Means Differences analysis with means and standard errors for Motif (*Q* = 2.37, *α* = 0.05) and Frieze Type (*Q* = 2.96, α = 0.05).

**Table 1.** Tukey least squared means differences HSD test with means and standard errors for motif and frieze type in Experiment 1.

Motif		Mean	Standard error
Texture	A	3.60	0.04
Comma	B	3.38	0.03
Flags	B	3.32	0.03
Frieze type		Mean	Standard error
TRHVG	A	4.06	0.06
THG	B	3.71	0.06
TRVG	C	3.36	0.05
TV	C	3.35	0.05
TG	C	3.29	0.05
TR	C	3.28	0.05
T	D	3.01	0.04

*Note*. Levels not connected by the same letter are significantly different. TRHVG = translation, 180° rotation, horizontal mirror, vertical mirror, and glide reflection; THG = translation, horizontal mirror, and glide reflection; TRVG = translation, 180° rotation, vertical mirror, and glide reflection; TG = translation plus glide reflection; TR = translation and 180° rotation; TV = translation plus vertical mirror.

We next performed a set of planned comparisons by contrasting conditions against one another. This was done in order to answer a set of questions regarding motif and frieze. The results are shown in Table 2 which shows the kind of contrast, the purpose and the derived conclusion. Degrees of freedom for all of these contrasts is always one. The *t* and associated *p* values are shown. Textured friezes were liked more than the combined mean of friezes containing flags and commas. In every case where one or more symmetries are added to a frieze containing a single symmetry, the frieze with the greater number of symmetries is preferred.

**Table 2.** Planned contrasts with goals and purpose for Experiment 1.

Mean contrast	Questions and conclusions
Tx vs. FC	To see if texture friezes are preferred to those with a single motif (a flag or a comma)
*t* = 6.01, *p* < .01	Conclusion: Yes
T vs. TG	To see if glide reflection improves preference when added to friezes containing translation only
*t* = 3.78, *p* < .01	Conclusion: Yes
T vs. TR	To see if rotation improves preference when added to friezes containing translation only
*t* = 3.61, *p* < .01	Conclusion: Yes
T vs. TV	To see if vertical mirrors improve preference when added to friezes containing translation only
*t* = 4.31, *p* < .01	Conclusion: Yes
TG vs. THG	To see if horizontal mirrors improve preference when added to friezes containing translation and glide reflection
*t* = 5.63, *p* < .01	Conclusion: Yes
TRVG vs. TRHVG	To see if horizontal mirrors improve preference when added to friezes containing translation, rotation, vertical mirrors and glide reflection.
*t* = 9.46, *p* < .01	Conclusion: Yes
THG vs. TRHVG	To see if two symmetries (rotation and vertical mirrors) improve preference when added to friezes containing translation, horizontal mirrors and glide reflection
*t* = 4.70, *p* < .01	Conclusion: Yes

*Note*. Motif legend: Tx = Texture, F = Flag, C = Comma.

Frieze type legend: T = Translation, G = Glide reflection, R = Rotation, V = Vertical mirror, H = Horizontal mirror. TRHVG = translation, 180° rotation, horizontal mirror, vertical mirror, and glide reflection; THG = translation, horizontal mirror, and glide reflection; TRVG = translation, 180° rotation, vertical mirror, and glide reflection; TG = translation plus glide reflection; TR = translation and 180° rotation; TV = translation plus vertical mirror.

### Discussion

The results show a clear preference for friezes with filled regions. The textures that filled the square block areas were considered more beautiful than the commas or flags. In addition, the THG and TRHVG friezes that occupied the entire frieze region were liked the most. One explanation for this effect is that filled patterns are more likely to form emergent features. These are collections of small black squares that form together to create larger features, most likely by the gestalt laws of grouping such as proximity, collinearity, and good continuation, etc. These features in turn make the symmetries in the pattern more noticeable, which may enhance their perceived beauty. Although we attempted to reduce the formation of such features by introducing spacing near the borders, they remain within the interior regions of the texture blocks.

There was no difference between the comma and flag motif. Both were deliberately equated in terms of size and overall shape but varied with respect to curvature to see if this affected responding. Prior research shows a preference for curved shapes (Cotter et al., 2017). This suggests that for these types of motifs it seems to be the symmetrical relationship between the objects rather than the characteristics of the objects themselves that influence perceived beauty. What observers find aesthetically pleasing are the correpondences or mappings between the motifs instead of the static individual properties of each motif shape.

In terms of rank ordering pattern, TRHVG was liked the most. This pattern contains the greatest number of symmetries. At the other end of the spectrum pattern T which contains the fewest symmetries was liked the least. However, the results do not show a strict complexity ordering effect. If this were the case pattern TRVG would have been liked second since it contains the second greatest number of symmetries. Instead pattern THG was liked second, probably because it is a completely filled pattern and also contains emphatic horizontal mirrors. In this case, the emergent features created by the filled pattern seem to outweigh symmetric complexity. Overall the results show only a partial preference for patterns with a greater number of symmetry operations. Table 3 shows the rank order predictions based on the complexity in comparison to the obtained results.

**Table 3.** Rank ordered predictions for friezes based on number of symmetries compared to actual outcomes in Experiment 1.

Predicted
1. TRHVG (5 symmetries)
2. TRVG (4 symmetries)
3. THG (3 symmetries)
4. TG, TR, TV (2 symmetries)
5. T (1 symmetry)
Obtained, based on significance
1. TRHVG (Highest ranking)
2. THG (2nd-highest ranking)
3. TRVG, TV, TG, TR (3rd-highest ranking)
4. T (lowest ranking)

TRHVG = translation, 180° rotation, horizontal mirror, vertical mirror, and glide reflection; THG = translation, horizontal mirror, and glide reflection; TRVG = translation, 180° rotation, vertical mirror, and glide reflection; TG = translation plus glide reflection; TR = translation and 180° rotation; TV = translation plus vertical mirror.

One can also examine different friezes with regard to the types of symmetries they contain using the addition approach. For instance, by looking at TV, TG, and TR in relation to T (translation only), we can see whether the addition of any of these three individual symmetries changes beauty ratings. It was the case that adding vertical mirrors, glide reflection, and rotation to a frieze with only translation does make the pattern more appealing. Increased preference ratings were also found when horizontal mirrors were added to a frieze with translation and glide reflection or to a frieze with translation, rotation, vertical mirrors, and glide reflection. Similarly, we can compare TRHVG to THG (an addition of R and V) to see if adding rotation and vertical mirrors to translation, horizontal mirrors, and glide reflection makes a difference. The results show that this also increased the pattern's appeal. So in every case where we add one or two extra symmetries to a frieze with a given number of symmetries, ratings go up significantly.

The only exception, as noted above, was TRVG (all symmetries except horizontal mirrors) because it was liked the same as TV, TG, and TR, so the combination of all of these symmetries together in one frieze did not improve ratings relative to when they were each present in isolation. Horizontal symmetry as we noted seems to improve ratings, perhaps because it emphasizes or runs parallel to the overall pattern orientation. The results show that the number of symmetries by themselves cannot explain their perceived beauty until other factors like the degree of fill or emergent features are accounted for.

There was an interaction between Motif and Frieze Type. An examination shows a higher mean responding for TRHVG and THG for textures compared to commas and flags. As discussed above, this is likely because these completely fill the pattern region and perhaps also because they contain horizontal symmetry. There are also secondary more minor elevations for T and TV as well. These patterns fill half the pattern region, so this can explain why they are liked comparatively more for textures also. The remaining texture conditions (TG, TR, and TRVG) have alternating blocks that fill the top and bottom regions of the frieze. This alternation may break up the overall pattern or makes the symmetries less easy to discern. This may explain why TRVG was preferred so little, even though it contained the second greatest number of symmetries.

## Experiment 2

In Experiment 1 filled texture patterns were preferred over individual motifs. In addition, when comparing textured friezes to one another, those that were completely filled (top and bottom of the frieze) were preferred more than those that were partially filled, being either one half of the frieze, or alternating filled blocks in the top and bottom half. Some of the patterns in the first experiment were filled and some were not. This makes some comparisons difficult. We don’t know in certain instances whether it was the type of symmetry or the fill that can account for perceived beauty. In order to correct for this we performed a second experiment in which all of the friezes were textured and filled.

The use of completely filled patterns allows us to vary the size and number of elements in the texture. We can for example create friezes with a smaller number of large elements or a larger number of small elements. Increasing the number of elements in the pattern is one way to increase complexity. But it also appears to make discriminating the symmetries between blocks in the patterns more difficult. Friedenberg and Liby (2016) filled fixed square areas with different levels of smaller black squares. Ratings peaked for mid-level densities that corresponded to maximim levels of complexity as measured by a GIF compression ratio. In the current study, we decided to vary the complexity of each texture block by subdividing it into either a more subdivided (16 × 16 array) or less subdivided (4 × 4) set of squares than those used in experiment 1 (which were 8 × 8). The 4 × 4 division produces a smaller number of larger squares and an ostensibly simpler pattern. The 16 × 16 division produces a larger number of smaller squares and an ostensibly more complex pattern. If element number translates into complexity and this is preferred then we should see an increase in ratings with array size. If symmetric discriminability affects ratings, then we might expect the opposite of this effect, with preference for patterns that have fewer elements.

### Method

#### Participants

Fifty-one undergraduates participated in order to fulfill a class requirement. There were 24 males and 27 females. Vision of all participants was normal or corrected to normal. Average age of the students was 19.84 years. All participants voluntarily agreed to participate and signed a consent form. American Psychological Association standards were followed.

#### Stimuli

**Figure 4.**
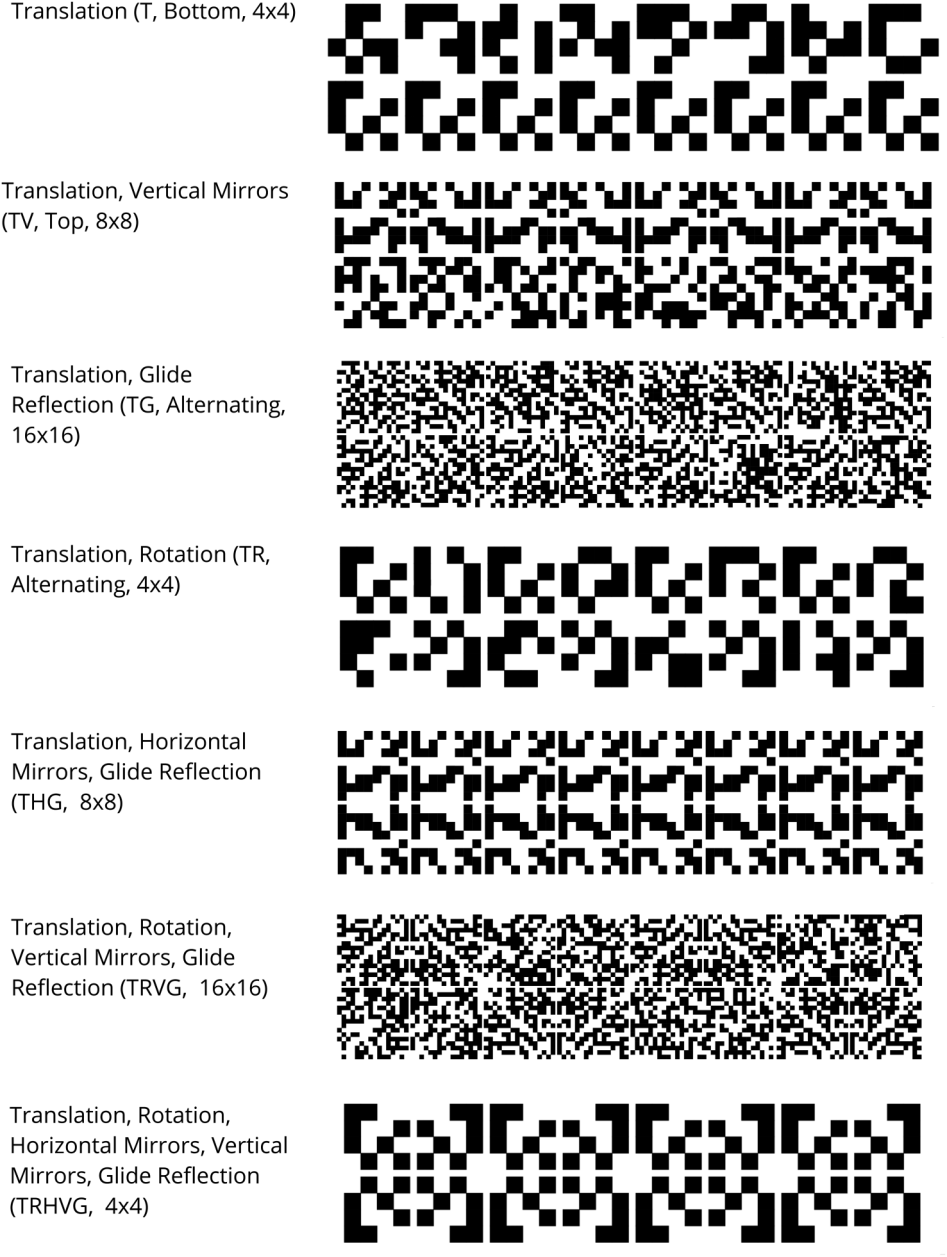
Selected examples of each of the different friezes used in Experiment 2.

The seven frieze types were again used. However, there were different variants for each this time depending upon how the signal and noise blocks were distributed across the frieze region. A signal block (S) was a 50% random fill that was repeated by a symmetry operation. This signal block pattern was constant across frieze types for each array size condition to ensure uniformity of comparison. A noise block (N) was also a 50% random fill but was different for every block where it occurred across all patterns. To illustrate, the T frieze could have a repeating motif across its top half and noise across its bottom (SSSSSSSS-NNNNNNNN) or the inverse (NNNNNNNN-SSSSSSSS). Frieze TG which is an alternating pattern could have two phase-shifted versions of itself starting with signal in the top half (SNSNSNSN-NSNSNSNS) or noise in the top half (NSNSNSNS-SNSNSNSN). Complete variants for each frieze type are shown in Table 4. Representative examples of each pattern are shown in Figure 4.

**Table 4.** Frieze type versions and pattern codes for Experiment 2.

Frieze type	Version (Variant)	Pattern code
T	Top	SSSSSSSS-NNNNNNNN
	Bottom	NNNNNNNN-SSSSSSSS
TV	Top	SSSSSSSS-NNNNNNNN
	Bottom	NNNNNNNN-SSSSSSSS
TG	SNSN	SNSNSNSN-NSNSNSNS
	NSNS	NSNSNSNS- SNSNSNSN
TR	SNSN	SNSNSNSN-NSNSNSNS
	NSNS	NSNSNSNS- SNSNSNSN
THG	Filled	SSSSSSSS-SSSSSSSS
TRVG	SSNN	SSNNSSNN-NNSSNNSS
	NNSS	NNSSNNSS- SSNNSSNN
TRHVG	Filled	SSSSSSSS-SSSSSSSS

*Note*. S = Signal (Same random noise block for all patterns within a size array and which is repeated by the symmetry operation), N = Noise (A random noise block that is always different).

Letters before dash indicate top half of pattern, letters after dash indicate bottom half of pattern. TRHVG = translation, 180° rotation, horizontal mirror, vertical mirror, and glide reflection; THG = translation, horizontal mirror, and glide reflection; TRVG = translation, 180° rotation, vertical mirror, and glide reflection; TG = translation plus glide reflection; TR = translation and 180° rotation; TV = translation plus vertical mirror.

Each of the conditions above was presented in three array sizes. The size of the friezes and the size of the block regions within them were the same as in experiment 1. The size of the small black and white squares in the 4 × 4 condition was .62 cm, in the 8 × 8 condition it was 0.31 cm, and in the 16 × 16 condition it was 0.15 cm. The friezes were again presented as black patterns against a white background.

#### Procedure

Although there are 12 signal-noise conditions as shown in Table 4, THG and TRHVG were presented twice to equate the number of variants per frieze type in a block. This resulted in 14 conditions multiplied by three array sizes to produce 42 trials in a block. Each participant was shown ten blocks for a total of 420 trials in an experiment session. Trial sequence within a block was randomized. Trial duration was response terminated. A session took about 20 min to complete. Stimuli were presented at a standard viewing distance of about 48 cm. Beauty rating judgments (Likert scale of 1–7) were obtained for each trial using the number keys running across the top of the keyboard. All other response conditions were identical to those of the first study.

### Results

**Figure 5.**
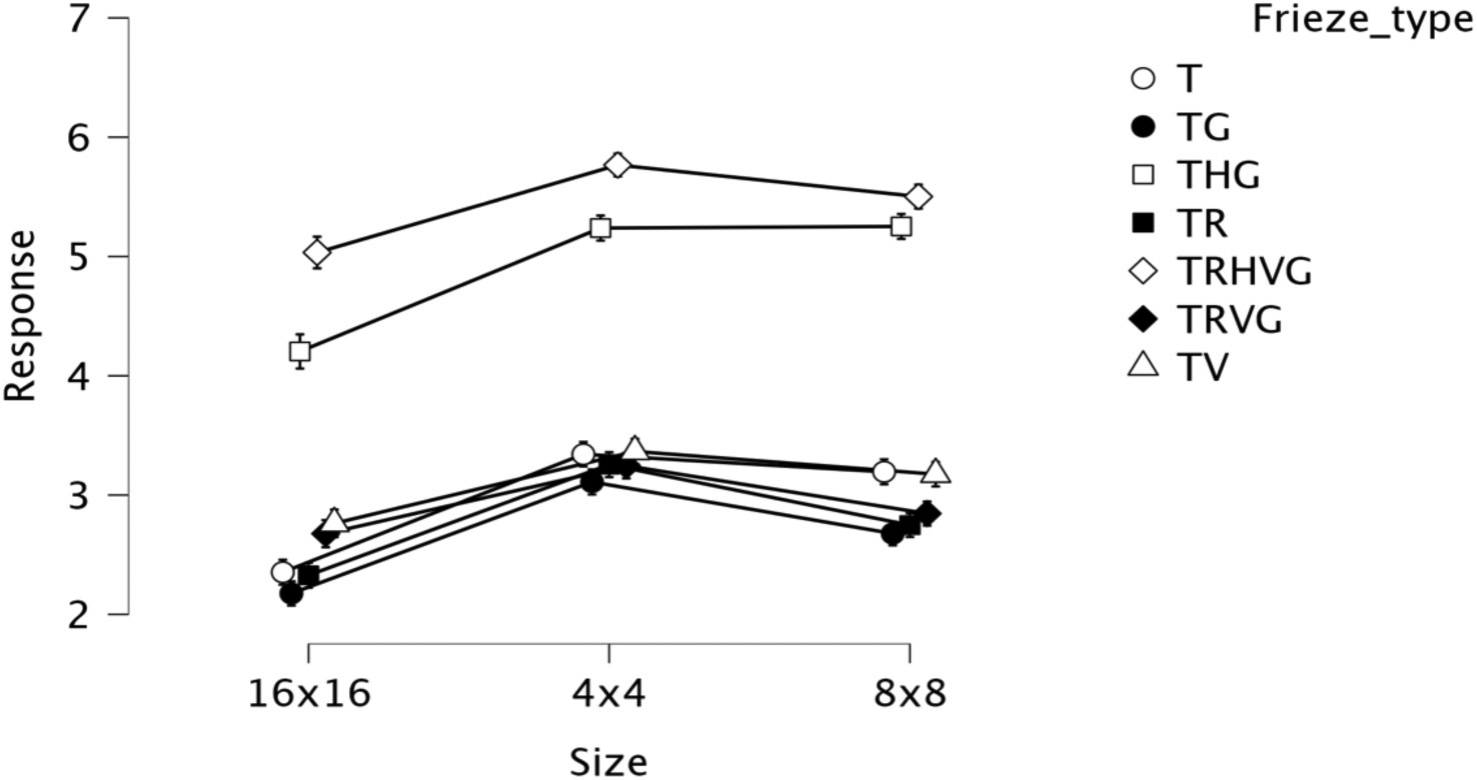
Mean and 95% confidence interval plots for the main effects of size, frieze type and their interaction from Experiment 2.

A two-way ANOVA with alpha level set at 0.05 yielded a main effect for Size (4 × 4, 8 × 8, 16 × 16), *F* (2, 150) = 412.95, *p* < .001 (η^2^_p_ = 0.44), and Frieze Type (T, TR, TV, TG, THG, TRVG, and TRHVG), *F* (6, 350) = 1,280.61, *p* < .001, (η^2^_p_ = 0.88). Additionally, there was a significant interaction between Size and Frieze Type, *F* (12, 600) = 8.68, *p* < .001, (η^2^_p_ = 0.09). Figure 5 contains line plots showing the effects of the interaction. Friezes containing the smallest number of large elements were liked the most (4 × 4), followed by those with intermediary number/size values (8 × 8). Friezes with the greatest number of smallest elements (16 × 16) were liked the least. Frieze TRHVG was liked the most, followed by THG. Table 5 shows the Tukey HSD least square means and standard errors of the conditions for size (*Q* = 2.37, α = 0.05) and frieze type (*Q* = 2.93, α = 0.05) ordered by statistical differences.

**Table 5.** Tukey least squared means differences HSD test with means and standard errors for element size and frieze type in Experiment 2.

Size		Mean	Standard error	
4 × 4 (Large)	A	3.90	0.02	
8 × 8 (Medium)	B	3.62	0.02	
16 × 16 (Small)	C	3.07	0.03	
Frieze type		Mean	Standard error	
TRHVG	A	5.43	0.03	
THG	B	4.89	0.04	
TV	C	3.10	0.03	
T	C D		2.96	0.03
TRVG	D E	2.92	0.03	
TR	E F	2.77	0.03	
TG	F	2.65	0.03	

*Note*. Levels not connected by the same letter are significantly different. TRHVG = translation, 180° rotation, horizontal mirror, vertical mirror, and glide reflection; THG = translation, horizontal mirror, and glide reflection; TRVG = translation, 180° rotation, vertical mirror, and glide reflection; TG = translation plus glide reflection; TR = translation and 180° rotation; TV = translation plus vertical mirror.

We note here that in the original analysis for both experiments the data was transformed by the formula of (rating − minimum score)/(maximum score − minimum score) × 100 in order to make the range of the scale more interpretable. We acknowledge that the use of tests like ANOVA for this type of rating scale data may be inappropriate because the assumptions require parametric data, which is not the case for a Likert scale. In order to determine if this transformation affected the results, we reanalyzed all of the results in both experiments using the raw rating scale data (untransformed). This did not affect any of the differences between conditions or statistical significance in the *F*-tests. It also did not affect any of the subsequent mean comparisons. The plots and tables showing the main effects and interactions were also unchanged as were the results of the planned contrasts performed in Experiment 1. Based on this we conclude that transforming the ratings at least for our studies did not affect any of the outcomes. Both the raw data, analyses of the results with the untransformed data and examples of the stimuli have been uploaded to Open Science Framework and can be found at this link: https://osf.io/amv6f/

### Discussion

There was a clear preference in this study for friezes with larger and less numerous element sizes. Preference peaked for the larger 4 × 4 arrays and decreased in a linear manner as elements got smaller and more numerous. If element density corresponds to complexity then the data indicate a preference for simpler patterns. However, simpler patterns also make the symmetric transformations in the friezes more discriminable. It is easier to see which texture blocks map onto one another when the features are larger in the 4 × 4 condition. One must more closely scrutinze the 8 × 8 and 16 × 16 blocks to see the symmetric correspondences. Perhaps this is because the larger elements appear as parts with distinct features. One can see translated, reflected, and rotated versions of these larger features across the blocks and compare them more readily. Smaller features, especially those in the 16 × 16 condition, appear less as distinct parts and more like aspects of a surface.

If we compare the results for frieze type across the two studies we see some similarities and some differences. First, we replicated the finding that TRHVG and THG are the two most preferred pattern types, with TRHVG being liked the most and THG second-most in both experiments. This seems to be a robust effect, with these two conditions much more highly elevated than any of the other friezes regardless of motif or element size. These are the only two friezes that contain horizontal mirrors, suggesting that this kind of symmetry plays a pivotal role. All of these friezes were presented at horizontal orientations, so horizontal symmetry runs parallel to the orientation of the frieze itself. This parallelism may accentuate the overall pattern and could in turn facilitate detection of the other symmetries.

The ordering of the remaining conditions across the two experiments differs. In Experiment 1 TRVG (everything except horizontal symmetry) ranked third, making it the next best preferred after the friezes with horizontal mirrors. We attributed this to symmetric complexity, since this pattern contains four symmetries. However, in Experiment 2 frieze TRVG fared worse, ranking only in fifth place. This suggests that horizontal symmetry is far more influential than the total number of symmetries in determining perceived beauty. Vertical mirrors on the other hand seem to exert less aesthetic power. Frieze TV for example ranked fourth in experiment 1 and third in experiment 2.

## General Discussion

We can derive a number of conclusions from this work. For the stimuli we tested, friezes with a random texture are liked more than those containing distinct individual motifs. Texture elements group together and form larger elements (what we call emergent features) that may enhance symmetries and make them easier to detect. Textures are also more complex visually and so may be preferred when paired against single isolated elements. However in the second study when all of the patterns were textured, the most complex ones with the smallest elements were liked least. It may be that there is a limit to how small an element can get and still be seen as a feature. The 16 × 16 cases were quite small and may have been perceived as a texture or surface rather than as features or objects. If this were true then it may have masked any of the symmetries present.

In Experiment 1 there was no difference between the use of angular (a flag) or curved motifs (a comma). This result however does not imply that motif properties never affect frieze aesthetics. Larger motifs that fill up the available space more might compete for textures in terms of greater preference. One could manipulate motif size, adjacency, color, fill, texture, and other characteristics to determine their potential influence. In particular, one could produce motifs that emphasize or de-emphasize specific symmetries to see if this enhances ratings.

Our initial prediction was that visual patterns with a greater number of symmetries would be liked more. Friezes are a good test case for this as they vary systematically in the number and type of symmetries they contain. Symmetry is a type of regularity and prior research shows a regularity preference (Bertamini & Rampone, 2020). Our results show that the frieze that was liked the most contained the greatest number of symmetries, but a rank ordering of the results based on the degree of regularity does not match up to prediction.

In Experiment 2 we equated all patterns in terms of texture and varied both the number and size of textural elements. This was done by carving up a basic frieze block into smaller and smaller array units. Since some friezes completely use up the rectangular space available and others only fill half of it, we additionally created all possible versions with top and bottom halves and alternating empty blocks filled with random noise. We found that observers prefer friezes with larger and fewer elements because they might enhance the ease with which symmetric correspondences can be perceived. To confirm this explanation further research is needed. One could ask participants to perform regularity detection or symmetry recognition tasks, and then look at the extent to which these measures correlate with aesthetic judgments.

There is further potential for the investigation between perceptual grouping, symmetry and aesthetic evaluation. The current study suggests that emergent features facilitate the perception of visual symmetries and that this in turn enhances subjective beauty. In Experiment 2, however, there was a confounding of element number and size. When element features became larger they also became less numerous. It is possible though to create larger emergent features while holding number constant. This can be achieved by using dots that group increasingly by proximity but that do not touch one another. The introduction of such textures into friezes holds element number (i.e., unit density) constant while varying the strength of larger feature-like representations. These visual stimuli could then be used to test for both regularity detection, detection of specific individual symmetries and perceived beauty.

One consistent finding across both experiments was that the top two choices were frieze TRHVG (all symmetries including horizontal) as number one and frieze THG (horizontal plus translation and glide reflection) as number two. These are the only two friezes that have horizontal mirrors. We suspect that horizontal mirrors are important because they run parallel to the overall frieze orientation. This may make the symmetries that are present more obvious or salient. It may be the case that there is nothing special about horizontal symmetry in an absolute sense. There are studies showing horizontal visual symmetry is actually less easily detected than vertical visual symmetry (Baylis & Driver, 1994; Kahn & Foster, 1986). One way to test this is to rotate horizontal friezes so that they appear in a vertical orientation.

The study of friezes is a first step toward a broader understanding of decorative art (Gombrich, 1994). Although ornament has been studied in a qualitative way, it deserves much greater empirical attention. Most ornamental decoration consists of motifs mapped onto one another across two-dimensional space by symmetric transforms. The current study demonstrates that these operations affect the perceived beauty of such patterns in a predictable way. The continued study of symmetry and its associated factors will help to unlock the secrets of these enduring and alluring patterns.
